# Passive smoking as a risk factor for dementia and cognitive impairment: systematic review of observational studies

**DOI:** 10.1017/S1041610217002824

**Published:** 2017-12-18

**Authors:** Lucy E. Stirland, Chris I. O'Shea, Tom C. Russ

**Affiliations:** 1 Centre for Dementia Prevention, University of Edinburgh, Edinburgh, UK; 2 Postgraduate Education Centre, Royal Infirmary of Edinburgh, Edinburgh, UK; 3 Alzheimer Scotland Dementia Research Centre, Edinburgh, UK

**Keywords:** dementia, cognitive impairment, risk factors, environmental, passive smoking, systematic review

## Abstract

**Background:**

Smoking is a well-established risk factor for dementia, but the effects of passive smoking are unclear. We aimed to examine links between passive smoking and dementia or cognitive impairment.

**Methods:**

We searched seven medical research databases: MEDLINE, Web of Science (Core Collection), Cochrane, EMBASE, PsycINFO, Scopus, and CINAHL Plus. Studies were included if they examined measures of passive smoking and either cognitive impairment or dementia.

**Results:**

Of 1,425 records found, nine papers of varying methodologies were included after screening against inclusion criteria. Eight papers reported weak associations between passive smoking and either cognitive impairment or dementia. One paper only found this association alongside carotid artery stenosis. The papers’ quality was variable, with only two deemed high quality.

**Conclusion:**

There is limited weak observational evidence linking passive smoking with an increased risk of cognitive impairment or dementia. However, the studies were methodologically diverse and of inconsistent quality, preventing firm conclusions.

## Introduction

Dementia is increasing in worldwide prominence and has major public health and economic implications (Prince, 2015). Consequently, the prevention of dementia through addressing modifiable risk factors has become an important focus for research. Tobacco smoking is an accepted risk for dementia (Peters *et al.*, 2008), through vascular damage which is in turn linked to stroke, Alzheimer's disease, and other dementias. However, relatively little is known regarding “passive” smoking and dementia, though a recent systematic review of environmental factors for dementia included one article suggesting moderate evidence of an association with passive smoking (Killin *et al.*, 2016). Proposed mechanisms include vascular damage similar to that caused by smoking, interference with brain oxygenation, and neuroinflammation (Ghosh *et al.*, 2009; Ling and Heffernan, 2016).

Some of the existing literature refers to “cognitive impairment,” a term which can encompass a variety of clinical syndromes including Mild Cognitive Impairment and static deficits.

There have been no literature reviews to date on the links between passive smoking and dementia in adults. We, therefore, present the first systematic review on the association between passive smoking and both dementia and cognitive impairment.

## Methods

### Information sources

Two reviewers conducted a search of seven online databases: MEDLINE, Web of Science (Core Collection), Cochrane, EMBASE, PsycINFO, Scopus, and CINAHL Plus on 10th December 2014.

### Search

“Passive” smoking is also referred to as “second-hand smoke,” “environmental tobacco exposure,” or “involuntary smoking.” Its definition normally includes both smoke exhaled by a nearby smoker and “side-stream” smoke released into the environment by lit cigarettes or other smoked tobacco (McKenzie *et al.*, 2014). Therefore, our search terms included combinations of keywords for passive smoking (passive, second-hand, environmental, and involuntary smoking) and dementia, cognitive impairment, cognitive function, or Alzheimer's disease. Full search terms are in Appendix A1, available as supplementary material online attached to the electronic version of this paper at http:/journals.cambridge.org/ipg. Anticipating the field of research to be small, we intentionally kept our search terms broad and inclusive. We included studies with cognitive impairment or dementia as the outcome of interest and set no age limit in adults.

We augmented this search by tracking citations of each of the papers using Google Scholar, and examining the bibliographies of each paper for relevant titles.

### Study selection and eligibility criteria

Two reviewers independently screened the emergent titles against inclusion and exclusion criteria and produced a list of abstracts, which were further assessed for relevance before obtaining the full articles for selected records. The inclusion criteria specified that papers must contain measures of both exposure to passive smoking and cognitive function or dementia. Both longitudinal and cross-sectional studies were included. Papers were excluded if they examined cognitive impairment in children, current smokers only, or passive smoking among smokers. Non-English language articles were also excluded, as were non-human studies. Papers consisting solely of systematic review were excluded.

### Data collection process

Having gathered our list of included papers two authors (LS, CO) independently used a standardized data extraction form to collect key process and outcome data. We collated these summary data and resolved any disagreements.

### Risk of bias within and between studies

We created a tailored quality assessment tool with the aim of detecting selection bias, confounding, and information bias (Hammer *et al.*, 2009). For example, we looked for clearly defined outcomes measured using objective criteria. Using this tool, we separately assessed each paper's quality and risk of bias before agreeing together a final overall quality score: high, satisfactory, or low. High quality papers were considered to have little or no risk of bias and to contain objective measures of cognitive function and passive smoking. Satisfactory quality was defined as showing some suggestion of flaws in the study which could lead to a risk of bias. Low quality papers were considered to have significant flaws in key aspects of study design which could bias the results.

We aimed to assess publication bias if possible. We excluded papers which duplicated work published elsewhere, for example articles reporting similar analyses within a single dataset or cohort.

### Registration

This review was prospectively registered with the University of York's Centre for Reviews and Dissemination PROSPERO register on 22nd September 2014 (record number CRD42014013543). Ethical approval was not sought as we did not conduct any primary research.

## Results

### Study selection

**Figure 1.**
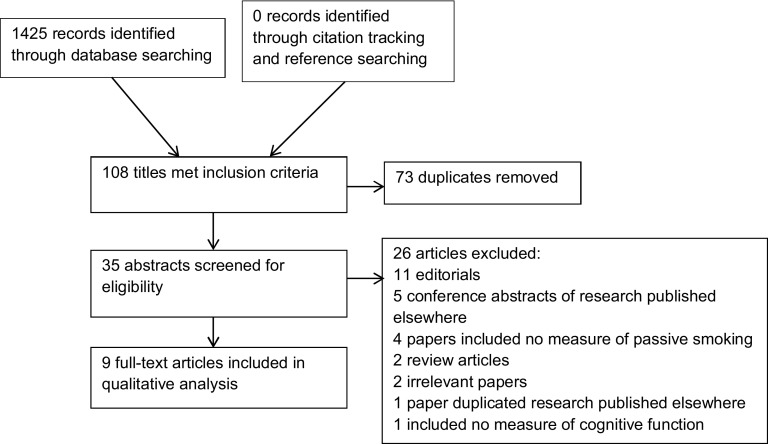
PRISMA flow diagram of systematic review search process.

Our search identified a total of 1,425 records from the databases listed in Table 1. Figure 1 depicts a PRISMA diagram summarizing the search, screening, and selection process. No additional papers were identified through citation tracking and bibliography searching. After removal of duplicates, 35 titles met the inclusion criteria. These abstracts were then screened for eligibility and 26 excluded, for reasons stated in Figure 1.

**Table 1.** Databases searched

database	year database started	number of titles found
MEDLINE	1946	114
Web of Science (Core Collection)	1900	25
Cochrane	1993	34
EMBASE	1980	150
PsycINFO	1806	23
Scopus	1970	1,005
CINAHL Plus	1937	74

### Synthesis of results

The studies differed in their methodology, reported outcomes, and statistical methods. We considered the possibility of meta-analysis and found it would not be appropriate to pool data for this reason (The Cochrane Collaboration, 2011). A summary of our rationale in Appendix A2, available as supplementary material online attached to the electronic version of this paper at http:/journals.cambridge.org/ipg. We have summarized each paper's methods, findings, and our overall quality ratings in Table 2.

**Table 2.** Synthesized summary of papers reporting the association between exposure to passive smoking and cognitive impairment or dementia

study	methodology and location	sample description	measurement of passive smoking	measurement of cognitive functioning	confounders measured	significant findings	quality score
Akhtar *et al*. (2013)	Cross-sectional. Community-based sample in USA.	*n* = 2,452 non-smokers aged ≥ 60, 1,489 exposed to passive smoke* baseline rate of dementia not given.	Defined as serum cotinine level 0.011– 9.53 ng/mL.	DSST, self-reported functional limitation due to confusion and memory problems.	Age, race/ethnicity, gender, education, diabetes, hypertension, BMI, stroke, heart attack, and alcohol use.	Cotinine level had no significant association with self-reported confusion and memory problems. Minimally adjusted linear regression (95% CI) of cotinine level in people who had never smoked was associated with a change in DSST score by −2.03 (−3.00, −1.05). When fully adjusted the change in DSST score was −1.17 (−2.32, −0.02).	H
Llewellyn *et al*. (2009)	Cross-sectional. Community-based sample in England.	*n* = 4,809. Mean age 65. Proportion exposed to passive smoke not given. A total of 481 cognitively impaired at baseline.	Defined as salivary cotinine level 0.0–14.1 ng/ml, split into fourths.	Composite scores on: Letter Cancellation Task, MMSE (time orientated questions), Health and Retirement Study 10 Word Learning Task, Prospective Memory Tasks, and simple calculations, CAMCOG - Semantic Verbal Fluency. Cognitive impairment defined as lowest 10% of summarized scores.	Several models of adjustment. Fully adjusted model: age, sex, education, testing interval, ethnicity, manual occupation, net wealth, smoking history, obesity, alcohol consumption, physical inactivity, depressive symptoms, and medical conditions (diabetes, cardiovascular disease, stroke, treated, and untreated hypertension).	Compared with lowest fourth of cotinine concentration (0.0–0.1 ng/mL), multi-variable adjusted odds ratios (95% CI) for cognitive impairment in the highest fourth (0.8–13.5 ng/mL) was 1.70 (1.03, 2.80) among never smokers. Associations between lower cotinine levels and cognitive impairment were non-significant. In the fully adjusted model, there was a significant trend of cognitive impairment with increasing cotinine concentration (*p* for trend 0.025).	H
Barnes *et al.* (2010)	Longitudinal. Four communities in USA.	*n* = 970 never smokers with no baseline cardiovascular disease, mean age 74. A total of 500 lived with a smoker, none had dementia diagnosis at baseline.	Self-report. Number of years living with smoker, categorized into three levels of exposure.	Follow-up at mean 5.5 years to assess progression to dementia. Dementia diagnosis made by multi-professional adjudication committee based on cognitive tests scores (Modified MMSE), hospital records, ADL ability, and other clinical factors.	Age, race, gender, income, education, Apolipoprotein-E genotype, CRP, occupation, self-reported health, hypertension, diabetes, physical activity, depression, weight, cholesterol, and alcohol intake.	Using Cox proportional hazards with marginal structural models, there was no evidence of association between SHS exposure alone and dementia. Adjusted hazard ratio (95% CI) of dementia with >25 years’ SHS exposure and >25% carotid artery stenosis was 3 (1.03, 9.72).	S
Chen (2012)	Cross-sectional. Four separate urban and rural areas in China (Four province study).	*n* = 2,692 never smokers, mean age 72, 810 exposed to passive smoke 49 had dementia syndrome at baseline.	Self-report. Split into “none at all;” “yes, some,” and “yes, a lot” for three different environments; reported years of exposure.	GMS-AGECAT, Modified CERAD, and CSI-D. Those with scores in the top 15% were interviewed clinically to establish diagnosis.	Age, sex, urban/rural location, education level, occupation, income, marital status, religion, current drinking, visiting children or relatives, head injuries, COPD, hypertension, and stroke.	Multivariable Cox-adjusted Risk Ratio (95% CI) for all dementia was 1.78 (1.18, 2.69). Increased exposure at home led to further increased risk.	S
Chen *et al*. (2012) (Research Letter)	Cross-sectional. Four separate urban and rural areas in China (Four province study, women only)	*n* = 1,979 female never smokers aged ≥60. A total of 635 exposed to passive smoke, 174 had dementia syndrome at baseline.	Self-report. Split into “none at all;” “yes, some,” and “yes, a lot” for three different environments; reported years of exposure.	GMS-AGECAT; case level score ≥3. Scores 1 and 2 labelled “subcases.”	Age, province, urban/rural location, educational level, occupation, marital status, COPD, head injuries, hypertension, and stroke.	Multivariable Cox-adjusted Risk Ratio (95% CI) for cognitive impairment with SHS exposure 1.39 (1.01, 1.89), increasing with self-reported dose and duration. No association found between exposure to SHS and subcases.	S
Chen *et al*. (2013a)	Cross-sectional. Rural and urban areas in China (third wave of Anhui study).	*n* = 1,081 never smokers, mean age 76, 350 exposed to passive smoke, 166 cases of dementia at baseline.	Self-report. Split into “none at all;” “yes, some,” and “yes, a lot” for three different environments; reported years of exposure.	GMS-AGECAT; case-level score ≥3.	Age, sex, BMI, urban/rural location, education level, occupational class, marital status, religion, head injury, hypertension, diabetes, stroke, COPD, fish and vegetable consumption	Multivariable adjusted Risk Ratio (95% CI) for cognitive impairment was statistically significant when cumulative years of exposure was ≥50 (RR 1.82, 95% CI 1.20, 2.76), increasing with dose and duration.	S
Chen *et al*. (2013b)	Cross-sectional. Five separate urban and rural areas in China (combination of four province study and third wave of Anhui study).	*n* = 5,921. Mean age 73. A total of 2,153 exposed to passive smoke, and 626 had dementia syndrome at baseline.	Self-report as “none at all;” “yes, some,” and “yes, a lot” for three different environments; reported years of exposure.	GMS-AGECAT; case-level score ≥3 defined as “severe dementia syndrome” (scores 1 and 2 defined as “moderate dementia syndrome”).	Age, sex, urban/rural location, education level, occupation, smoking status, income, marital status, religion, current drinking, visiting children or relatives, hypertension, stroke, and depressive syndrome.	Multivariable adjusted Risk Ratio (95% CI) for “severe dementia” with SHS exposure in never smokers 1.33 (1.01, 1.74) increasing with self-reported dose and duration. No association between exposure to SHS and “moderate dementia syndrome.”	S
Orsitto *et al*. (2012)	Cross-sectional. Inpatients in geriatric ward, Italy.	*n* = 933, mean age 77, 96 exposed to passive smoke. A total of 124 had dementia at baseline.	Self-report. Structured questionnaire from patient or relative. Number of hours of smoke exposure in past seven days.	MMSE, CDR. Dementia and MCI diagnoses made clinically according to international diagnostic criteria.	Age, educational level, and smoking history.	Multivariable-adjusted odds ratio (95% CI) for MCI in those exposed to SHS compared to dementia 2.7 (1.5, 4.4) and compared to cognitively intact individuals 1.9 (1.0, 3.5).	S
Heffernan and O'Neill (2013)	Between-Groups Design. Psychology laboratory, England.	*n* = 79 students, mean age 23. A total of 24 were exposed to passive smoke, none had baseline cognitive impairment. Divided into groups: current smokers, non-smokers exposed to SHS, and non-smokers not exposed to SHS.	Self-report questionnaire: number of hours exposed to SHS per week.	CAMPROMPT	Age, alcohol consumption, HADS anxiety and depression score, and NART score.	ANCOVA analysis. There were significant differences between groups on the CAMPROMPT test, with the non-SHS group recalling significantly more time-based tasks than the SHS group (*p* < 0.001).	L

* = passive smoking taken as any detectable serum cotinine.

Quality scores: H = high, S = satisfactory, and L = low.

DSST = Digit Symbol Substitution Test.

BMI = Body Mass Index.

MMSE = Mini Mental State Examination.

CAMCOG = Cambridge Cognitive Examination.

SHS = Second-hand smoke.

CRP = C-reactive protein.

GMS-AGECAT = Geriatric Mental Status – Automated Geriatric Examination for Computer Assisted Taxonomy.

CERAD = Consortium to Establish a Registry for Alzheimer's Disease.

CSI-D = Community Screening Instrument for Dementia.

COPD = Chronic obstructive pulmonary disease.

CDR = Clinical Dementia Rating Scale.

CI = Confidence interval.

MCI = Mild cognitive impairment.

CAMPROMPT = Cambridge Prospective Memory Test.

HADS = Hospital Anxiety and Depression Score.

NART = National Adult Reading Test.

ANCOVA = Analysis of covariance.

PM = Prospective memory.

ADL = Activities of Daily Living.

### Study characteristics

Nine papers were included in the final analysis, four of which reported research conducted in China (Chen, 2012; Chen *et al.*, 2012; 2013a; 2013b), two in the UK (Llewellyn *et al.*, 2009; Heffernan and O'Neill, 2013), two in the USA (Barnes *et al.*, 2010; Akhtar *et al.*, 2013), and one in Italy (Orsitto *et al.*, 2012). They comprised seven cross-sectional studies, one between-groups design, and one longitudinal study. One paper presented a systematic literature review alongside new study data (Chen *et al.*, 2013a).

All studies included standard cognitive tests and justified their use. Three studies also included clinical interview to verify diagnoses of dementia or the presence of cognitive impairment (Barnes *et al.*, 2010; Chen, 2012; Orsitto *et al.*, 2012). One paper included both a standardized test and self-reported functional limitation from memory impairment (Akhtar *et al.*, 2013). Four studies (Barnes *et al.*, 2010; Chen, 2012; Chen *et al.*, 2012; 2013b) referred to dementia as a measured outcome, one included both dementia and Mild Cognitive Impairment (Orsitto *et al.*, 2012), another three focused on general cognitive impairment (Llewellyn *et al.*, 2009; Akhtar *et al.*, 2013; Chen *et al.*, 2013a), and one paper specifically examined prospective memory (Heffernan and O'Neill, 2013).

To quantify exposure to passive smoking, two studies measured levels of cotinine, a metabolite of nicotine, which is an accepted biological marker of exposure to cigarette smoke (Llewellyn *et al.*, 2009; Akhtar *et al.*, 2013). The remaining seven studies relied on self-report using different structured interview templates. No studies used a combination of biomarker and self-report.

Six papers reported an association between increased levels of passive smoking and a form of cognitive impairment (Llewellyn *et al.*, 2009; Chen *et al.*, 2012; Orsitto *et al.*, 2012; Akhtar *et al.*, 2013; Chen *et al.*, 2013a; Heffernan and O'Neill, 2013). Two papers described an association between passive smoking and dementia (Chen, 2012; 2013b). One paper reported no association between passive smoking alone and dementia, but found an association in a sub-group analysis of people with greater than 25 years’ of exposure to passive smoking and more than 25% carotid artery stenosis (Barnes *et al.*, 2010).

## Discussion

Most papers reported an association between passive smoking and either cognitive impairment or dementia. However, overall there was a paucity of evidence and the majority of studies were at moderate risk of bias. In particular, all of the studies specifically examining dementia were only of satisfactory quality, and the highest quality research was limited to cognitive impairment. The papers’ methodological heterogeneity prevented meta-analysis so we did not find convincing evidence of any associations.

Recent interest in this topic is reflected in the fact that all the papers identified were published since 2009, which complements contemporary research into the links between air pollution and dementia (Weuve *et al.*, 2012; Killin *et al.*, 2016; Chen *et al.*, 2017).

### Comparison with other literature

To our knowledge, there is only one other systematic review on the topic of passive smoking and cognitive impairment, presented in a 2013 paper alongside novel study data (Chen *et al.*, 2013a). It excluded studies where the endpoint was dementia on the questionable basis that this differs pathologically and prognostically from cognitive impairment. It used a lower age limit of 60 years and therefore only reviewed three papers. Therefore, ours is the first comprehensive systematic review of the associations between passive smoking and both dementia and cognitive impairment.

### Strengths and limitations

Our review sought to be comprehensive by using broad search terms in multiple databases with no age limits in adults; this is reflected in the wide range of records returned by the search. The fact that no papers were found using citation tracking or bibliography searching provides further evidence suggesting that our search method was exhaustive. We included studies reporting both dementia and cognitive impairment, which is a term used inconsistently to describe a variety of clinical states. There is a lack of standardized quality assessment tools for systematic reviews of this type. We, therefore, created our own quality assessment tool structured around common causes of bias and based on existing guidelines for evaluating cohort studies (Hammer *et al.*, 2009; Scottish Intercollegiate Guideline Network, 2012).

Our restriction to English-language publications may have led to the exclusion of potentially relevant research findings in other languages. Although the databases we searched were predominantly in English, some papers in other languages were excluded at the title screening stage.

Overall, because outcome measures differed, it was impossible to directly compare the strengths of association between studies and to quantitatively meta-analyze the results. Therefore, conclusions from our review are limited to general observations on cognitive test results or diagnostic outcomes.

### Measurement of passive smoking

A number of studies identified passive smoking through self-reporting which is open to criticism. In the studies based in China, high illiteracy rates (reported as 61% in one paper (Chen *et al.*, 2013a)) may have influenced participants’ answers to the question “have you experienced passive smoking”? The author goes on to explain that *“*most Chinese people are unaware of the health hazards of either active or passive smoking,” with only 32% of people understanding that exposure to passive smoking carries health risks. Furthermore, there was variation between the papers’ implied definitions of exposure to passive smoking. For example, one paper included structured questionnaires with more detail about exposure to cigarette smoke in different environments but this only applied to the last seven days (Orsitto *et al.*, 2012). Conversely, another study asked participants about exposure to cigarette smoke at home across the lifespan. However, this study did not include workplace or other sources of second-hand smoke (Barnes *et al.*, 2010). This variation in measurement of passive smoking was one of the factors limiting our ability to meta-analyze all the studies’ findings.

Two papers used cotinine levels as a biomarker for passive smoking. Cotinine has a half-life of 15–19 hours (Benowitz, 1996), which means that cross-sectional measurement of this only reflects exposure to second-hand smoke within recent days. If passive smoking were considered a risk factor for a chronic neurodegenerative disease such as dementia, care should be taken not to infer lifetime exposure from a single biomarker measurement and repeated measures would be likely to give a more accurate estimate of actual exposure. Given that the neuropathological processes underlying dementia are present long before the onset of symptoms (Price and Morris, 1999), it would be intuitive to combine both biomarkers of recent exposure and self-reported historical exposure. This method is suggested as the most reliable measure of passive smoking (Pérez-Ríos *et al.*, 2013) and we would recommend it be used in future studies.

### Measurement of cognitive impairment and dementia

Four of the nine included papers were by the same first author and sampled different combinations of study participants from the same two cohorts. All of these papers identified cognitive impairment using the GMS-AGECAT algorithm. However, there were variations in how the categories were interpreted across the studies. For example, a score of 3 or more was defined as a “severe dementia syndrome” in one study (Chen *et al.*, 2013b) and as “caseness” in others (Chen *et al.*, 2012; 2013a). Severe dementia suggests a different clinical picture than mere “caseness” and this inconsistency could lead to difficulty in interpreting these results and applying them in clinical practice.

The outcome measures for cognitive function across the studies varied. In the papers exploring general cognitive impairment rather than dementia, outcomes included several different neuropsychological tests, with or without clinical assessment and participants’ subjective experience of memory loss. One study used a prospective memory test, which may be less relevant to clinical dementia assessment (Heffernan and O'Neill, 2013). Only one paper specifically investigated Mild Cognitive Impairment as an outcome but its cross-sectional design did not allow for measuring progression to dementia (Orsitto *et al.*, 2012). The three papers where dementia was the outcome also had diverse outcomes, with one including scores on a cognitive test alone, one adding an unspecified clinical interview procedure, and the third one combining a cognitive test and the opinion of a multi-professional adjudication committee.

### Risk of bias within and between studies

None of the studies included or mentioned power calculations. Most of the studies were of relatively large cohorts and the analyses may have been adequately powered but this was not formally discussed in the papers. None of the papers mentioned whether raters were blinded when deciding outcomes. In cases where a clinical diagnosis was made, no papers included clarification of whether this was repeated by a second professional.

Publication bias suggests that studies finding positive associations are more likely to be published. This could have led to the disproportionate representation of positive results in the literature we found (Guyatt *et al.*, 2011). The diversity of the papers’ outcomes and statistical methods prevented us from calculating publication bias or preparing a funnel plot.

A potential source of confounding is the smoking history of participants. All studies acknowledged this but accounted for it in different ways, either by excluding former smokers, analyzing them separately or adjusting for smoking history in their analyses. In addition, people exposed to passive smoking may share lifestyle factors with smokers, adding further potential confounding (Koo *et al.*, 1997). Four studies adjusted for alcohol intake, two considered physical activity, and one referred to specific dietary factors.

Four of the nine papers studied combinations of participants from two cohort studies. These did not meet our exclusion criteria because each paper studied a different subset with some variation in methods and were not strictly duplications. We included and discussed all of these for transparency. One of the papers (Chen *et al.*, 2013b) contained participants which all appeared in three of the other papers (Chen, 2012; Chen *et al.*, 2012; 2013a). There is, therefore, overlap between participants analyzed. This is a major source of bias and, given the relatively small field of research, affects the overall generalizability of the work we reviewed.

In the analysis of some studies’ results, ordinal data were used to make numerical calculations. This involved passive smoking being categorized as “no, none at all,” “yes, some,” or “yes, a lot” and each of these groups being allocated an “exposure level” number, 0, 1, or 2. The number of self-reported years of exposure to second-hand smoke was then multiplied by these “exposure levels” to give what the authors called a cumulative dose. The authors then performed calculations that inferred an increasing risk of dementia syndromes with increasing cumulative dose (Chen, 2012; Chen *et al.*, 2012; 2013a; 2013b). Calculating risk and odds ratios using ordinal data in this manner could be seen as introducing analytical bias and confers a significant limitation to the affected studies.

Our broad search strategy led to the inclusion of a study assessing the association of passive smoke on changes in prospective memory in a sample of undergraduate students (Heffernan and O'Neill, 2013). There was a limitation in this study, which may make interpretation of its findings difficult. Its exclusion criteria included *“*drinking in excess of UK Government guidelines for safe drinking*”* but the mean alcohol consumption across the groups studied was 30–34 units per week. At the time of the paper's publication, this level of intake exceeded UK Government recommendations on sensible drinking (Department of Health, 2007). We corresponded with the author who explained that reference to this exclusion criterion was an error. In addition, the exclusion criteria included participants who had a current psychiatric condition. However, the mean Hospital Anxiety and Depression Scale (HADS) scores of the second-hand smoke group was 9.62 which is a borderline abnormal result, which could indicate abnormal levels of anxiety and depression, a potentially important confounding factor.

### Potential mechanisms

All the studies we included were observational and therefore, cannot be used to infer causality; the possibility of residual confounding remains. However, evidence from animal and human studies supports a mechanistic link between tobacco smoking and cognitive impairment and dementia, probably due to microglial activation and neuroinflammation (Ghosh *et al.*, 2009; Moreno-Gonzalez *et al.*, 2013). Passive smoking is considered a risk factor for other diseases such as stroke and cardiovascular disease because it causes vascular changes common to those caused by smoking (Glantz and Parmley, 1995; Jefferis *et al.*, 2010). These vascular effects are possible mechanisms to explain links between passive smoking and dementia or cognitive impairment in later life (Ling and Heffernan, 2016).

There is some evidence to suggest a link between exposure to passive smoking and poorer cognitive outcomes in children and adolescents (Yolton *et al.*, 2005). Speculative hypotheses for why this might be the case include the effect of carbon monoxide from tobacco smoke interfering with brain oxygenation (Ling and Heffernan, 2016). There is also evidence from mouse models that procarcinogens in tobacco smoke cause neuroinflammation, particularly in the hippocampus, which is responsible for aspects of learning and memory (Ghosh *et al.*, 2009).

Other possible explanations for the associations found could be confounding factors, which have not yet been identified as risk factors for dementia and were therefore not accounted for. Only two of the papers we reviewed were graded as high quality, defined as having little or no risk of bias. Therefore, this may also have contributed to the direction of overall findings.

## Conflict of interest

None.

## Description of authors’ roles

L Stirland designed data collection and quality assessment tools, screened for relevant articles, extracted data, assessed papers' quality, and wrote the paper. C O'Shea extracted data, assessed papers' quality, and wrote the paper. T Russ conceived of the research question, supervised data collection, and edited the paper.

## Supplementary material

For supplementary material accompanying this paper visit http://dx.doi.org/10.1017/S1041610217002824. click here to view supplementary material

## References

[ref001] Akhtar W. Z., Andresen E. M., Cannell M. B. and Xu X. (2013). Association of blood cotinine level with cognitive and physical performance in non-smoking older adults. Environmental Research, 121, 64–70. 23199696 10.1016/j.envres.2012.10.013 PMC3864778

[ref002] Barnes D. E., Haight T. J., Mehta K. M., Carlson M. C., Kuller L. H. and Tager I. B. (2010). Secondhand smoke, vascular disease, and dementia incidence: findings from the cardiovascular health cognition study. American Journal of Epidemiology, 171, 292–302. 20051462 10.1093/aje/kwp376 PMC2878108

[ref003] Benowitz N. L. (1996). Cotinine as a biomarker of environmental tobacco smoke exposure. Epidemiologic Reviews, 18, 188–204. 9021312 10.1093/oxfordjournals.epirev.a017925

[ref004] Chen H. et al (2017). Living near major roads and the incidence of dementia, Parkinson's disease, and multiple sclerosis: a population-based cohort study. The Lancet, 389, 718–726. 10.1016/S0140-6736(16)32399-6 28063597

[ref005] Chen R. et al (2013b). Association between environmental tobacco smoke exposure and dementia syndromes. Occupational & Environmental Medicine, 70, 63–69. 23104731 10.1136/oemed-2012-100785 PMC3534257

[ref006] Chen R. L. (2012). Association of environmental tobacco smoke with dementia and Alzheimer's disease among never smokers. Alzheimer's & Dementia, 8, 590–595. 10.1016/j.jalz.2011.09.231 22197095

[ref007] Chen R., Hu Z., Orton S., Chen R. L. and Wei L. (2013a). Association of passive smoking with cognitive impairment in nonsmoking older adults: a systematic literature review and a new study of Chinese cohort. Journal of Geriatric Psychiatry & Neurology, 26, 199–208. 23877565 10.1177/0891988713496165

[ref008] Chen R., Zhang D., Chen Y., Hu Z. and Wilson K. (2012). Passive smoking and risk of cognitive impairment in women who never smoke. Archives of Internal Medicine, 172, 271–273. 22332161 10.1001/archinternmed.2011.762

[ref028] Deeks J. J., Higgins J. P. T. and Altman D. G. (eds.). (2011). Chapter 9: Analysing data and undertaking meta-analyses. In J. P. T. Higgins and S. Green (Eds.), *Cochrane Handbook for Systematic Reviews of Interventions*, Version 5.1.0 (updated March 2011). The Cochrane Collaboration. Available at http://handbook-5-1.cochrane.org/chapter_9/9_analysing_data_and_undertaking_meta_analyses.htm.

[ref009] HM Government (2007). Safe. Sensible. Social. The next steps in the National Alcohol Strategy. London: Home Office.

[ref010] Ghosh D., Mishra M. K., Das S., Kaushik D. K. and Basu A. (2009). Tobacco carcinogen induces microglial activation and subsequent neuronal damage. Journal of Neurochemistry, 110, 1070–1081. 19500213 10.1111/j.1471-4159.2009.06203.x

[ref011] Glantz S. A. and Parmley W. W. (1995). Passive smoking and heart disease: mechanisms and risk. Journal of the American Medical Association, 273, 1047–1053. 7897790

[ref012] Guyatt G. H. et al (2011). GRADE guidelines: 5. Rating the quality of evidence-publication bias. Journal of Clinical Epidemiology, 64, 1277–1282. 21802904 10.1016/j.jclinepi.2011.01.011

[ref013] Hammer G. P., du Prel J. B. and Blettner M. (2009). Avoiding bias in observational studies: part 8 in a series of articles on evaluation of scientific publications. Deutsches Ärzteblatt International, 106, 664–668. 19946431 10.3238/arztebl.2009.0664 PMC2780010

[ref014] Heffernan T. M. and O'Neill T. S. (2013). Exposure to second-hand smoke damages everyday prospective memory. Addiction, 108, 420–426. 22913297 10.1111/j.1360-0443.2012.04056.x

[ref015] Jefferis B. et al (2010). Cotinine-assessed second-hand smoke exposure and risk of cardiovascular disease in older adults. Heart, 96, 854–859. 20478864 10.1136/hrt.2009.191148 PMC2921288

[ref016] Killin L. O. J., Starr J. M., Shiue I. J. and Russ T. C. (2016). Environmental risk factors for dementia: a systematic review. BMC Geriatrics, 16, 175. 27729011 10.1186/s12877-016-0342-y PMC5059894

[ref017] Koo L. C., Kabat G. C., Rylander R., Tominaga S., Kato I. and Ho J. H. (1997). Dietary and lifestyle correlates of passive smoking in Hong Kong, Japan, Sweden, and the USA. Social Science & Medicine, 45, 159–169. 9203280 10.1016/s0277-9536(96)00331-0

[ref018] Ling J. and Heffernan T. (2016). The cognitive deficits associated with second-hand smoking. Frontiers in Psychiatry, 7, 46. 27047401 10.3389/fpsyt.2016.00046 PMC4805605

[ref019] Llewellyn D. J., Lang I. A., Langa K. M., Naughton F. and Matthews F. E. (2009). Exposure to secondhand smoke and cognitive impairment in non-smokers: national cross sectional study with cotinine measurement. British Medical Journal, 338, b462. 10.1136/bmj.b462 PMC2643443 19213767

[ref020] McKenzie J., Bhatti L. and d'Espaignet E. T. (2014). WHO Tobacco Knowledge Summaries: Tobacco and Dementia. Geneva: World Health Organization.

[ref021] Moreno-Gonzalez I., Estrada L. D., Sanchez-Mejias E. and Soto C. (2013). Smoking exacerbates amyloid pathology in a mouse model of Alzheimer's disease. Nature Communications, 4, 1495. 10.1038/ncomms2494 23422663

[ref022] Orsitto G., Turi V., Venezia A., Fulvio F. and Manca C. (2012). Relation of secondhand smoking to mild cognitive impairment in older inpatients. Scientific World Journal, 2012, 726948. 22666146 10.1100/2012/726948 PMC3361321

[ref023] Pérez-Ríos M. et al (2013). Questionnaire-based second-hand smoke assessment in adults. The European Journal of Public Health, 23, 763–767. 22683770 10.1093/eurpub/cks069

[ref024] Peters R., Poulter R., Warner J., Beckett N., Burch L. and Bulpitt C. (2008). Smoking, dementia and cognitive decline in the elderly, a systematic review. BMC Geriatrics, 8, 36. 19105840 10.1186/1471-2318-8-36 PMC2642819

[ref025] Price J. L. and Morris J. C. (1999). Tangles and plaques in nondemented aging and “preclinical” Alzheimer's disease. Annals of Neurology, 45, 358–368. 10072051 10.1002/1531-8249(199903)45:3<358::aid-ana12>3.0.co;2-x

[ref026] Prince M., Wimo A., Guerchet M., Ali G. C., Wu Y. and Prina A. M. (2015). World Alzheimer Report 2015: The global impact of dementia. *An analysis of prevalence, incidence, costs and trends.* London, Alzheimer's Disease International.

[ref027] Scottish Intercollegiate Guidelines Network (SIGN). (2012). Methodology checklist 3: cohort studies, version 3.0. Edinburgh: SIGN. Available at http://www.sign.ac.uk/checklists-and-notes.html.

[ref029] Weuve J., Puett R. C., Schwartz J., Yanosky J. D., Laden F. and Grodstein F. (2012). Exposure to particulate air pollution and cognitive decline in older women. Archives of Internal Medicine, 172, 219–227. 22332151 10.1001/archinternmed.2011.683 PMC3622279

[ref030] Yolton K., Dietrich K., Auinger P., Lanphear B. P. and Hornung R. (2005). Exposure to environmental tobacco smoke and cognitive abilities among U.S. Children and adolescents. Environmental Health Perspectives, 113, 98–103. 15626655 10.1289/ehp.7210 PMC1253717

