# Supplementary material for: Passive smoking as a risk factor for dementia and cognitive impairment: systematic review of observational studies
Source: Int Psychogeriatr. 2017 Dec 18;30(8):1177–87. doi: 10.1017/S1041610217002824 (PMC6128010; doi:10.1017/S1041610217002824)
Supplement: Supplementary file 1 [file S1041610217002824sup001.docx]

**Appendix A2**

**Summary of attempt to pool data in meta-analysis**

Of the nine included papers, the methods used to measure exposure and outcome variables differed. There were six different combinations of cognitive or diagnostic tests and four methods of measuring passive smoking (two based on the biomarker cotinine and two on self-report questionnaires). The studies’ designs varied, with seven cross-sectional, one longitudinal and one laboratory-based between-groups design. The outcomes were reported using five different statistical models. One paper attracted a “low” score on our quality rating scale, suggesting a high risk of bias. This study would therefore not be suitable for inclusion in a meta-analysis.

The diversity of these numerous factors, in addition to the small number of papers found, led us to conclude that meta-analysis would not be appropriate (The Cochrane Collaboration, 2011).

See Table A1 for a summary of the included papers.

Table A1. Summary of studies’ characteristics

| **Study** | **Methodology** | **Measurement of passive smoking** | **Measurement of cognitive functioning** | **Statistical measure reported** | **Quality Score** |
| --- | --- | --- | --- | --- | --- |
| Akhtar *et al*., 2013 | Cross-Sectional | Serum cotinine level 0.011 - 9.53 ng/mL | DSST, self-reported functional limitation | Linear regression | High |
| Llewellyn *et al*., 2009 | Cross-Sectional | Salivary cotinine level 0.0 - 14.1 ng/mL | Composite scores on six cognitive tests | Odds ratios | High |
| Barnes *et al.*, 2010 | Longitudinal | Self-report. Number of years living with smoker | Dementia diagnosis made by multi-professional adjudication committee using modified MMSE and clinical information | Cox proportional hazards with marginal structural models | Satisfactory |
| Chen, 2012 | Cross-Sectional | Self-report categorical | GMS-AGECAT, modified CERAD, CSI-D | Multivariable Cox-adjusted risk ratio | Satisfactory |
| Chen *et al*., 2012 (Research Letter) | Cross-Sectional | Self-report categorical | GMS-AGECAT | Multivariable Cox-adjusted risk ratio | Satisfactory |
| Chen *et al*., 2013a | Cross-Sectional | Self-report categorical | GMS-AGECAT | Multivariable adjusted risk ratio | Satisfactory |
| Chen *et al*., 2013b | Cross-Sectional | Self-report categorical | GMS-AGECAT | Multivariable adjusted risk ratio | Satisfactory |
| Orsitto *et al*., 2012 | Cross-Sectional | Self-report. Number of hours of smoke exposure in past 7 days | MMSE, CDR. Dementia and MCI diagnoses made clinically | Multivariable-adjusted odds ratio (95% CI) | Satisfactory |
| Heffernan and O’Neill, 2013 | Between-Groups Design | Self-report questionnaire: number of hours of smoked exposure per week. | CAMPROMPT | ANCOVA analysis | Low |

DSST= Digit Symbol Substitution Test

MMSE= Mini Mental State Examination

GMS-AGECAT= Geriatric Mental Status – Automated Geriatric Examination for Computer Assisted Taxonomy

CERAD= Consortium to Establish a Registry for Alzheimer's Disease

CSI-D= Community Screening Instrument for Dementia

CDR= Clinical Dementia Rating Scale

MCI= Mild cognitive impairment

CAMPROMPT= Cambridge Prospective Memory Test

**References**

**The Cochrane Collaboration** (2011). Chapter 9, Analysing data and undertaking meta-analyses. In J. P. Higgins and S. Green (Eds.) Cochrane handbook for systematic reviews of interventions [www.handbook.cochrane.org](http://www.handbook.cochrane.org).
